# Estimating experienced racial segregation in US cities using large-scale GPS data

**DOI:** 10.1073/pnas.2026160118

**Published:** 2021-11-10

**Authors:** Susan Athey, Billy Ferguson, Matthew Gentzkow, Tobias Schmidt

**Affiliations:** ^a^Department of Economics, Stanford University, Stanford, CA 94305;; ^b^National Bureau of Economic Research, Cambridge, MA 02138;; ^c^Stanford Graduate School of Business, Stanford University, Stanford, CA 94305

**Keywords:** racial segregation, isolation, mobility

## Abstract

We estimate a measure of segregation, experienced isolation, that captures individuals’ exposure to diverse others in the places they visit over the course of their days. Using Global Positioning System (GPS) data collected from smartphones, we measure experienced isolation by race. We find that the isolation individuals experience is substantially lower than standard residential isolation measures would suggest but that experienced isolation and residential isolation are highly correlated across cities. Experienced isolation is lower relative to residential isolation in denser, wealthier, more educated cities with high levels of public transit use and is also negatively correlated with income mobility.

## Significance

Racial segregation shapes key aspects of a healthy society, including educational development, psychological well-being, and economic mobility. As such, a large literature has formed to measure segregation. Estimates of racial segregation often rely on assumptions of uniform interaction within some fixed time and geographic space despite the dynamic nature of urban environments. We leverage Global Positioning System data to estimate a measure of segregation that relaxes these strict assumptions. Experienced segregation according to our measure is substantially lower than standard measures would suggest. By decomposing segregation by functions of a city, like entertainment, restaurants, and retail, we facilitate targeted policy making where segregation matters most.

Social outcomes are profoundly shaped by the extent to which groups are segregated from one another (1–4). As a result, large literatures have developed in economics, sociology, and related fields seeking to measure the extent of segregation across space and time.

Most of this empirical work focuses on segregation in where people live. A leading measure is the isolation index, which captures the share of individuals’ neighbors who come from their own group (for example, refs. 1 and 5–7). If we view the object of interest as the exposure of one group to another (5, 8, 9), residential measures have obvious limitations. Individuals living in highly segregated neighborhoods may be exposed to diverse others where they work, shop, and socialize, while those living in apparently mixed neighborhoods may have little contact with their neighbors and commute to highly segregated places. A corollary is that standard residential segregation measures are highly sensitive to the way in which neighborhood boundaries are defined (8, 10).

An important exception to this residential focus is a body of work in sociology characterizing the activity space of individuals—the set of places individuals encounter as they move through their everyday lives (ref. 11 has a recent literature review of the activity space literature). Researchers leverage surveys (12–14) and geolocation data (15–18) to characterize the activity space for particular groups of people and the degree to which activity spaces overlap across groups.

In this paper, we build on this work to estimate an activity-based measure of segregation for a large sample of US cities using Global Positioning System (GPS) data. This experienced isolation has the same form as the isolation index, but rather than assuming that individuals are exposed uniformly to those in their neighborhood of residence, it averages exposure over the places individuals actually visit over the course of their days. This measure does not depend on arbitrary neighborhood boundaries, and it takes explicit account of the diversity experienced away from home. It can capture individual-level heterogeneity within neighborhoods (9), and it can be disaggregated across times of day, locations, and activities, thus giving a richer picture of the forces that increase or decrease segregation.

Our main data are GPS signals from a sample of US smartphone users covering ∼5% of the US population in the first 4 mo of 2017. The data are obtained from a company that aggregates anonymous pings from a range of smartphone apps. We observe each device’s home location as well as the location of every ping by the device recorded in the data. We map these locations to a grid of geographic units ∼500 ft × 500 ft, known as geohash7s. The sample of individuals is not random but is reasonably close to representative along a number of dimensions, and it has sufficient coverage that we can correct for deviations from representativeness using sample weights. We use the movement patterns we observe to compute experienced racial isolation.

Because we do not observe an individual’s race directly, we define the two types whose segregation we study as individuals with homes in majority White geohash7s and individuals with homes in majority non-White geohash7s. We refer to these two groups as WDs (White home geohash7 devices) and NWDs (Non-White home geohash7 devices) for simplicity. The median shares White of majority White and non-White home geohash7s are 0.89 and 0.22, respectively. We discuss below the implications of using these geographic definitions in place of individual race, and we show robustness to an alternative strategy that imputes race at the individual level.

We present four main results. First, peoples’ experiences as captured by our measure are substantially less isolated from diverse others than traditional residential isolation would suggest. The average experienced isolation across all metropolitan statistical areas (MSAs) is 0.46, compared with the average residential isolation of 0.61.* This implies that the share of WD’s exposures to other WDs is 46 percentage points greater than the share of NWD’s exposures to WDs.

Second, experienced isolation and residential isolation across MSAs are highly correlated. The overall correlation of the two measures among the 366 MSAs in our sample is 0.86. Among the 50 most populous MSAs, Milwaukee, WI; Detroit, MI; and Cleveland, OH rank in the top five in both residential and experienced isolation. Portland, OR; Seattle, WA; and Raleigh, NC rank in the bottom five for both measures.

Third, the variation in experienced relative to residential isolation is systematic. Experienced isolation is relatively lower in MSAs with higher population density and public transit use, consistent with the view that urban areas facilitate diverse interactions (19). Experiences are also less isolated in MSAs with higher income and education and lower unemployment, possibly reflecting a role for social capital in reducing segregation (20). Finally, relative experienced isolation is negatively correlated with the Chetty et al. (21) measure of income mobility, consistent with both diverse interactions increasing mobility and with areas that facilitate opportunity also promoting diverse interactions.

Fourth, decompositions across time and space reveal the extent to which different activities increase or decrease segregation. Experienced isolation is lowest during the day and highest in the morning and evening. Experienced isolation in home neighborhoods is higher than residential measures would suggest, whereas experienced isolation outside of home neighborhoods is much lower. Isolation is lowest at entertainment, retail, and eating establishments, while time at locations like churches and schools is somewhat more isolated.

These findings have several broader implications. They suggest that standard measures understate the frequency of exposure to diverse others in the United States, and they highlight important forces such as commercial activity that increase it. They suggest that residential measures may nevertheless be a good proxy when the main goal is to assess relative levels of segregation across cities. Finally, they suggest nuances to keep in mind when assessing where the negative effects of segregation are likely to be largest. For example, local public goods such as schools or police services that are explicitly tied to residential boundaries may be more likely to be provided in segregated environments.^†^ Children, those who do not work, and others whose daily life is more tied to their local neighborhoods are even less likely to be exposed to diverse others than current measures would suggest. Policies that affect the spatial distribution of commercial or leisure activities, or the transportation cost of accessing these activities, may be important tools for changing the distribution of exposure.

An important limitation to keep in mind in assessing all such implications is that we can only observe when devices occupy the same geographic space, not actual interaction between individuals. Under our construction, a restaurant goer is just as exposed to the waiter or the cook in the kitchen as she is to the person sitting across the table. White (23) highlights this subtlety by distinguishing geographic segregation (the concept we measure) and sociological segregation (based on actual interactions). While Sunstein (24) and others argue that geographic segregation in this sense is of interest on its own,^‡^ there are many reasons to think that the kind of exposure with limited interaction that occurs in places like shops and restaurants may have less social benefit than more sustained interpersonal contact.

We also emphasize two other important limitations of our analysis. First, we have no direct information about the individuals whose devices we see in our data, and so, we define individual types based on the demographic composition of home geohash7s rather than individual race. This means we are targeting a slightly different concept than much of the prior literature on segregation. We discuss alternative approaches, including imputing race at the individual level, in *SI Appendix*. Second, our sample is not fully representative, and the geolocation information about any given device is sparse.

This paper builds on a large literature on measuring urban segregation. Important early work on both the definition and measurement of segregation includes Duncan and Duncan (25), Taeuber and Taeuber (26), White (23), and Massey and Denton (8, 27). Cutler et al. (5) provide a comprehensive analysis of segregation in US cities over the century from 1890 to 1990. Card et al. (28) study the dynamics of neighborhood tipping, and Allcott et al. (29) examine high- and low-income neighborhood proximity to supermarkets and health inequality.

Our work is most related to the growing activity space literature on racial segregation, particularly Wong and Shaw (12), Phillips et al. (17), and Sampson and Levy (22). Our measure is closely related to the extension of Wong and Shaw (12) of the exposure-based isolation index to activity beyond residential interaction. Phillips et al. (17) use geotagged Twitter data to investigate racial segregation in 50 major cities based on mobility flows between neighborhoods. Sampson and Levy (22) use the Phillips et al. (17) estimates to similarly find that residential segregation is highly correlated with activity-based segregation. We extend prior work in that literature in several respects.

First, we estimate activity-based racial segregation across a larger set of US cities (366) and a larger sample of individuals (> 17 million) than prior work. Second, we statistically estimate the correlation of city characteristics with the divergence between residential and activity-based measures of racial isolation. Third, we take seriously the concern Wong and Shaw (12) voice about how to incorporate length of activity in measuring exposure by introducing a set of weights and exploring the robustness of such choices. Fourth, we provide decompositions of racial segregation by hour of day and by geographic features of a city, such as parks, churches, and restaurants.^§^

Our work is also related to a broader literature using GPS or similar location data to study social interactions.^¶^ In particular, Moore and Reeves (35) use a small geolocation dataset to motivate the need for dynamic measurement of racial and ethnic segregation,^#^ and contemporaneous work by Moro et al. (37) uses large-scale mobility data to study patterns of experienced segregation by income.

## Data

### Geography

We follow the literature in characterizing segregation at the level of MSAs and in using census tracts to approximate neighborhoods within MSAs (we omit micropolitan statistical areas). The finest geographic unit in our analysis is the geohash7, which as mentioned above, is a unit of a grid roughly 500 feet square.^‖^ We use census blocks to impute geohash7 demographics. *SI Appendix*, Figs. S1–S3 illustrate the relative sizes of geohash7s, census blocks, and census tracts, focusing on an urban census tract and a rural census tract in Birmingham, AL.

We obtain information about the location of establishments and features of interest from two sources: InfoUSA and OpenStreetMaps (OSM). The 2015 InfoUSA US Businesses mailing list contains the names, addresses, industries, and latitude/longitude for 15.6 million businesses in the United States. We take from the full list all establishments that belong to the broad categories of “restaurants and bars,” “civil, social and religious organizations,” “accommodation,” “sports and recreation,” “entertainment,” and “retail” (*SI Appendix*, section S1 has our manual classification of North American Industry Classification System (NAICS) code into these categories) 2,368,216 places all in all. We match each establishment to the geohash7s that contain its latitude/longitude. From OSM, we extract polygon data for outdoor spaces, like parks, playgrounds, sports fields, and gardens, and educational institutions, like schools, kindergartens, universities, and colleges (*SI Appendix*, section S1.2 has details). We associate each OSM feature with all geohash7s that intersect the feature’s polygon. *SI Appendix*, Fig. S4 depicts geohash7s associated with civil, social, and religious organizations; education; outdoor spaces; and restaurants and bars in downtown Birmingham, AL.

Many geohash7s are labeled with multiple features. We assume pings in a device’s home geohash7 (defined below) are at home regardless of what other features are present. We assign all pings in nonhome geohash7s that contain transportation features to transportation (in *SI Appendix*, section S2.1, we show that our results are robust to both dropping pings in transportation features and dropping sequential pings that are traveling over 12 mph). All other pings are allocated uniformly across features present in the geohash7.

### GPS Device Movements

Our GPS data are provided by a company that collects anonymous location data from mobile applications on users’ smartphones. The sample is an unbalanced panel of GPS “pings” from more than 17 million devices spanning January to April 2017.^**^ Pings are logged whenever an application on a device requests location information. In some cases, this will be the result of a device actively using an application, such as for navigation or weather information, while in other cases, applications may request the information even while running in the background. Pings thus occur at irregular intervals. For each ping, we observe a time stamp, a device identifier, and the geohash7 in which the ping occurs. The data also contain the geohash7 of each device’s home, inferred probabilistically from the device’s nighttime and early-morning pings.

### Demographics

We impute geohash7 demographics from the 2010 census. We match each home geohash7 to the census tract that contains its centroid. This yields a matching tract for 99.53% of devices in our sample. We match each home geohash7 to all census blocks that overlap its area. This yields a match to at least one census block with nonzero population for 98.12% of devices. We assign demographics to each home geohash7 by taking an area-weighted average of the demographics of the overlapping blocks (we show robustness to alternative methods of demographic imputation in *SI Appendix*, section S2.2). We define the “White” population based on the census designation “White alone (non-Hispanic),” and we group all other census race groups in the category “non-White.”

We use data on MSA characteristics from the 2010 American Community Survey (ACS) and the 2010 decennial census. These variables include the MSA’s median age, education level, unemployment rate, median income, population density, and share of residents using public transit to get to work (*SI Appendix*, Table S4 shows a complete description and sources for census, ACS, and mobility variables). We also use economic mobility measures from Chetty et al. (21) indicating the share of individuals born to parents at the 25th percentile of the income distribution who make it to the top quintile for White and Black populations. We compute MSA-level mobility measures by averaging across counties weighting by White and Black county populations, respectively.

### Summary Statistics

We observe 17,730,615 devices with home locations identified in 7,292,623 distinct geohash7s. We match these home geohash7s to 72,785 census tracts and 6,186,564 census blocks. This matching procedure succeeds for 17,397,580 devices, the final sample used throughout the rest of the paper.

To assess the representativeness of the sample, we compare the average census tract demographics of devices in our sample to averages in the US population. We find that our sample is representative in terms of gender, age, and unemployment rate. We find that it slightly oversamples more educated and wealthy areas, with average median income across census tracts in our sample about a thousand dollars more than the US mean, and census tract poverty rate about a percentage point lower. We address this imbalance by weighting as shown in Eq. **4** (in *SI Appendix*, Fig. S7, we compare the MSA device weighted [using weights 4] share White with the true share White of each MSA and find that the device weights effectively recover the true demographic shares). Details of this comparison and summaries of the average activity levels of devices in our sample are shown in *SI Appendix*, Tables S2 and S3, respectively.

While our WD and NWD designations are not equivalent to individual race, they are highly correlated with it. The median share White in a device’s home geohash7 is 0.22 for NWDs and 0.89 for WDs. We plot the histogram of this share for both groups in *SI Appendix*, Fig. S8.

## Measure

### Definition

Consider a population of individuals indexed by *i* and a set of MSAs or other geographic areas of interest indexed by *a*. We collect each individual who is a member of one of two groups, which we denote *W* and *NW*. In our analysis below, *W* will be individuals from majority White geohash7s (WDs), and *NW* will be individuals from majority non-White geohash7s (NWDs). Each individual has a set of exposures to other individuals in area *a*. We let $e_{i}\in \left[0,1\right]$ denote the share of individual *i*’s exposures that are to members of group *W*.^††^

A general form of the isolation index for area *a* captures the difference between the average value of *e_i_* among individuals in the two groups (cf. ref. 6):

(1) $$I_{a}=\frac{1}{|W_{a}|}\sum_{i\in W_{a}}e_{i}-\frac{1}{|NW_{a}|}\sum_{i\in NW_{a}}e_{i}.$$

Here, *W_a_* and *NW_a_* are the sets of individuals making up the two groups in area *a*, and ∣·∣ denotes the size of these sets. This measure ranges from zero–no isolation, with average *e_i_* equal for the two groups–to one–perfect isolation, with *e_i_* = 0 for all i∈NW and *e_i_* = 1 for all i∈W.

The standard version of this measure is residential isolation, which is equivalent to Eq. **1** under the assumption that each individual is exposed uniformly to others in her neighborhood of residence ([8], [10], [38]). In practice, neighborhoods are typically defined to be census tracts. Letting c(i) denote *i*’s census tract of residence and letting *r_c_* denote the share of the residents of tract *c* who are in group *W*, residential isolation is given by^‡‡^

(2) $$RI_{a}=\frac{1}{|W_{a}|}\sum_{i\in W_{a}}r_{c\left(i\right)}-\frac{1}{|NW_{a}|}\sum_{i\in NW_{a}}r_{c\left(i\right)}.$$

Because this measure does not rely on any information other than the racial composition of each neighborhood, it can easily be computed using aggregate census data.

The measure we define, experienced isolation, instead assumes that *e_i_* is given by the composition of the individuals actually present in the locations that *i* visits over time. We index time by t∈[0,1] and consider a finite set of locations within area *a* indexed by *l*. We think of a location *l* as a specific place, such as a restaurant, workplace, or park, that is much smaller than a neighborhood. In our application, locations will be geohash7s. Letting l(i,t) denote *i*’s location at time *t* and letting s(l,t) denote the share of individuals in location *l* at time *t* who are from group *W*, experienced isolation is defined to be^##^

(3) $$\begin{array}{l} EI_{a}=\frac{1}{|W_{a}|}\sum_{i\in W_{a}}\int_{t=0}^{1}s\left(l\left(i,t\right),t\right)dt-\\ \frac{1}{|NW_{a}|}\sum_{i\in NW_{a}}\int_{t=0}^{1}s\left(l\left(i,t\right),t\right)dt. \end{array}$$

### Estimation

Estimating experienced isolation *EI_a_* would be straightforward if we observed continuous location data for all individuals. While our GPS dataset is rich, it still falls well short of this ideal. There are two key limitations. 1) We observe locations only when a device pings rather than continuously. 2) We only observe a sample of individuals, not the full population. We make several simplifying assumptions in order to address these limitations.

To address limitation 1, we first assume that the times when an individual *i* visits a location *l* are not systematically selected to be times when s(l,t) is unusually high or low. That is, letting ${\bar{s}}_{l}$ denote the overall expectation of s(l,t) over t∈[0,1], we have $E\left[s\left(l,t\right)|l\left(i,t\right)=l\right]={\bar{s}}_{l}$ for all *i*. Provided this assumption holds, the expectation of the term $\int_{t=0}^{1}s\left(l\left(i,t\right),t\right)dt$ is equal to ${\bar{S}}_{i}=\sum_{l}q_{il}{\bar{s}}_{l}$, where *q_il_* is the expected share of *i*’s time that is spent in location *l*. We further assume that the times at which we observe pings are a random sample from [0,1], so we can estimate *q_il_* and ${\bar{s}}_{l}$ by the shares of *i*’s pings that occur in location *l* and the share of all pings in location *l* that come from *W*, respectively.

Both of these are strong assumptions. The first would be violated, for example, if type *W* individuals tend to visit a particular park or restaurant in the morning, while type *NW* individuals tend to visit it in the evening. *SI Appendix*, section S2.4 relaxes this assumption by defining locations *l* at the location-hour level and yields very similar results to our baseline specification. The second would be violated if our data oversample periods in which the relative share of type *W* individuals is unusually high or low. In *SI Appendix*, section S2.3, we present robustness to an alternative specification allowing nonrandom weighting of pings across time.

To address limitation 2, we reweight home locations in our sample to match the distribution of the population in the 2010 census. Because our data are relatively sparse at the geohash7 level, we reweight by census tract. We define the weight for individual *i* to be

(4) $$\lambda_{i}=\frac{N_{c\left(i\right)}}{{\tilde{N}}_{c\left(i\right)}},$$

where *N_c_* is the census population of tract *c* and ${\tilde{N}}_{c}$ is the number of devices in our sample with home locations in tract *c*.

Combining these assumptions, we form an estimator of *S_i_* as follows. First, we form a leave-out estimate of ${\bar{s}}_{l}$:

(5) $${\hat{s}}_{l}^{-i}=\frac{\sum_{j\in P_{l}^{-i}\cap W}\lambda_{j}}{\sum_{j\in P_{l}^{-i}}\lambda_{j}},$$

where $P_{l}^{-i}$ is the set of pings associated with individuals other than *i* who visit location *l*, and we abuse notation by letting *λ_j_* denote the weight of the individual associated with ping *j*. We omit visits by *i* from this measure to avoid a severe small-sample bias that can arise when some locations have a small number of observed visits (41–43). Second, we estimate ${\bar{S}}_{i}$ by

$${\hat{S}}_{i}=\frac{1}{|P_{i}|}\sum_{j\in P_{i}}{\hat{s}}_{l\left(j\right)}^{-i},$$

where $P_{i}$ is the set of pings associated with *i* and l(j) is the location of ping *j*.

Finally, we estimate experienced isolation by

$${\hat{EI}}_{a}=\frac{1}{|W_{a}|}\sum_{i\in W}\lambda_{i}{\hat{S}}_{i}-\frac{1}{|NW_{a}|}\sum_{i\in NW}\lambda_{i}{\hat{S}}_{i}.$$

We estimate residential isolation as

(6) $$\hat{RI_{a}}=\frac{1}{|W_{a}|}\sum_{i\in W_{a}}\lambda_{i}{\hat{r}}_{c(i)}-\frac{1}{|NW_{a}|}\sum_{i\in NW_{a}}\lambda_{i}{\hat{r}}_{c(i)},$$

where ${\hat{r}}_{c}$ is the share of devices in our sample with home census tract *c* that are WDs. This differs from the residential isolation measure typically reported in the literature because the types we consider are WDs and NWDs rather than White and Black individuals and because we infer ${\hat{r}}_{c}$ from our device data rather than census data.

## Discussion

Our measure of experienced isolation considers an individual to be exposed to another if they are in the same location at the same time. This is what allows us to write Eq. **3** replacing the *e_i_* of Eq. **1** with the average of s(l,t) across space and time. The set of people who contribute to an individual’s exposure is, as discussed in the introduction, quite different from the set of people with whom an individual actually interacts. To the extent that we view actual interactions as the true object of interest, our measure can be seen as an approximation, which significantly improves on residential measures but may still over- or understate isolation to the extent that interactions within different geohash7s are relatively more or less segregated. We decompose isolation into features of a city, like schools, churches, and restaurants/bars, to help inform the kinds of interactions implied by physical presence in the same geographic space.

In our empirical analysis, we define the types *W* and *NW* to be WDs and NWDs–devices from majority White and non-White home geohash7s–rather than White and non-White individuals. This is a departure from prior literature on residential segregation, where the assumption of uniform exposure within neighborhoods makes it possible to compute segregation based on individual race (using aggregate race shares measured in census data).

Therefore, the target of our estimation is subtly different from the standard target. To gain some intuition for the difference, note that individual geohash7s are perfectly segregated between WDs and NWDs by construction, whereas they are less than perfectly segregated by individual race. As noted, the median WD lives in a home geohash7, which is 89% rather than 100% White, and the median NWD lives in a home geohash7, which is 78% rather than 100% non-White. We show below that this leads residential isolation between WDs and NWDs to be higher than between individual Whites and non-Whites. While the true level of segregation under our definition may be different, we expect the qualitative patterns we emphasize (e.g., the comparison of residential with experienced segregation) to be robust across alternative definitions.

As support for this, we report in *SI Appendix*, section S3 results using an alternative strategy where we impute race stochastically at the individual device level based on the composition of a home geohash7. This has the advantage of bringing our target concept closer to that in the prior literature. It has the disadvantage of introducing measurement error in the measure of a device’s type that could create a downward bias in experienced segregation estimates.^¶¶^ While this alternative does change the level of segregation as expected, we confirm that our main qualitative conclusions are indeed robust.

## Main Results

**Fig. 1.**
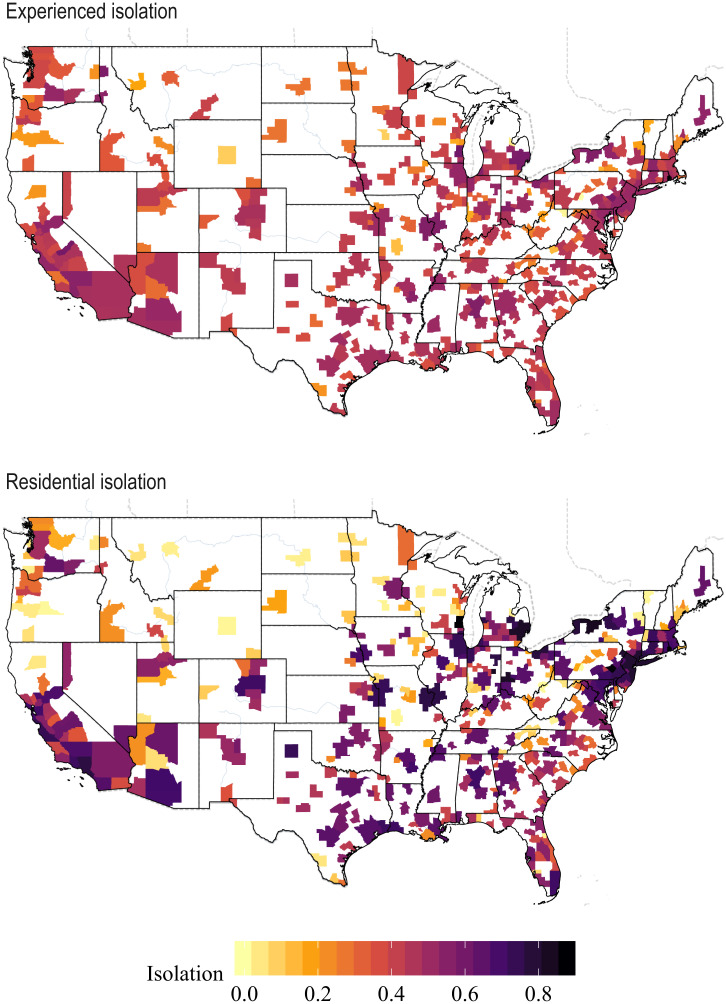
Experienced and residential isolation by MSA.

Fig. 1 shows estimated experienced and residential isolation for all MSAs in our sample (*SI Appendix*, Fig. S9 presents a map with the difference between experienced and residential isolation for each MSA, and *SI Appendix*, Tables S5–S7 report both experienced and residential isolation for each MSA). Two key facts are immediately apparent from these maps. First, experienced isolation is lower than residential isolation in large sections of the country. Second, the two measures are correlated across space, with both tending to be higher in the South, the Rust Belt, and in major cities and tending to be lower in the upper Midwest and Northwest.

**Fig. 2.**
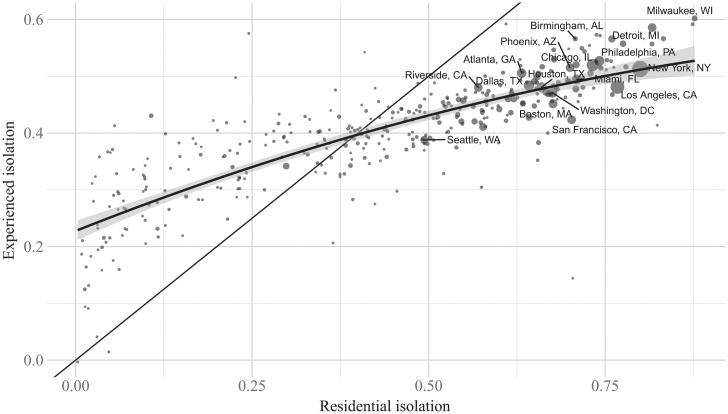
Experienced vs. residential isolation. The plot shows experienced and residential isolation for each MSA. The size of each point is proportional to the MSA’s population. The labeled points designate the 15 most populous MSAs. We plot the 45^∘^ line and a local polynomial fit.

Fig. 2 compares the two measures more directly, plotting experienced isolation against residential isolation. Experienced isolation is lower than residential isolation where residential isolation is high and higher than residential isolation where residential isolation is low. MSAs in the former category, however, account for the vast majority of the country’s population, including all 15 of the most populous MSAs, with 87.9% of people living in MSAs where experienced isolation is less than residential isolation. The population-weighted average experienced isolation across all MSAs is 0.46, compared with average residential isolation of 0.61. The 10th and 90th percentiles of experienced isolation are 0.37 and 0.53, respectively, compared with 0.34 and 0.78, respectively, for residential isolation. This figure also confirms that experienced isolation and residential isolation are highly correlated across MSAs, with a Pearson correlation coefficient of 0.864 and a Spearman rank correlation coefficient of 0.84. Among the 20 most populous MSAs, the ratio of experienced isolation to residential isolation is lowest (∼0.6) in San Francisco–Oakland–Fremont, CA and Los Angeles, CA and highest (∼0.8) in Atlanta, GA and Riverside, CA.

To describe the factors that correlate with lower experienced segregation, we regress experienced isolation on observed MSA characteristics controlling for 15 equal-sized bins of residential isolation. We focus on population-weighted univariate relationships, including a single observed characteristic in each case (*SI Appendix*, Table S8 shows similar results in regressions that are unweighted but subset to the top 50, 100, and 200 most populous MSAs). We emphasize that these are purely descriptive correlations and need not imply anything about the causes or effects of segregation.

**Fig. 3.**
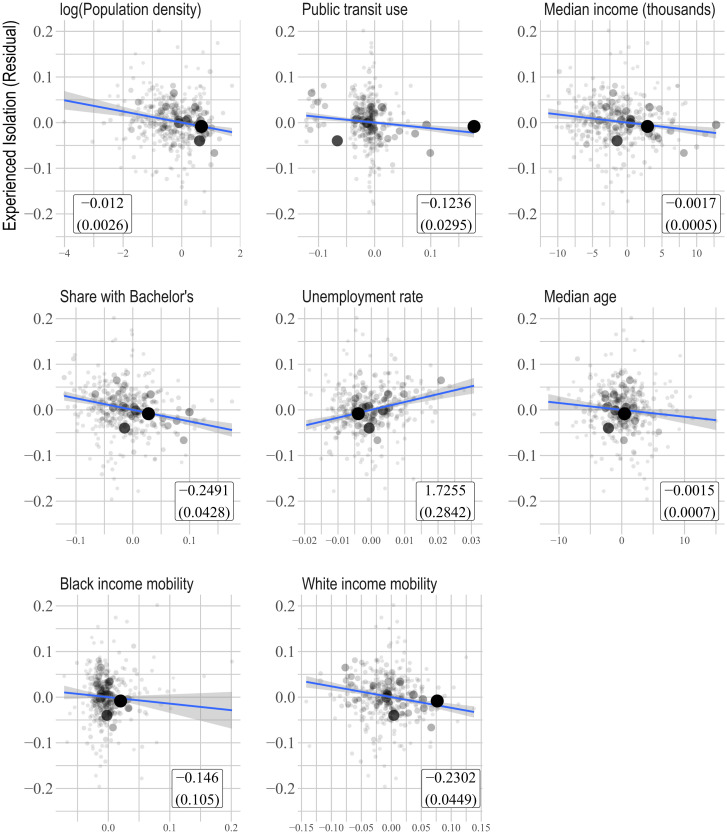
Residual experienced isolation and MSA characteristics. On the *y* axis, we plot the residual from a population-weighted regression of experienced isolation on 15 equal-sized bins of residential isolation at the MSA level. The *x* axis in each plot refers to the specified MSA characteristic. Each point refers to an MSA and is shaded and sized relative to total population. In the white boxes in the lower right corners, we show the coefficient and SE from the population-weighted regression of experienced isolation on the residential isolation bin fixed effects and the specified covariate. The blue lines show the population-weighted linear fits. The share with bachelor’s variable includes the percentage of people in an MSA who have at least a bachelor’s degree. The Black and White income measures average the Chetty et al. (21) county estimates (pooled by race) of the share of individuals born in the 25th percentile of the income distribution who make it to the top quintile. Public transit use is the share of the working population that uses public transport to get to work.

Fig. 3 shows the results. Each panel plots residuals of experienced isolation against residuals of a given MSA characteristic where the residuals are derived from regression on the residential isolation controls. Experienced isolation is relatively lower in MSAs with higher population density and more public transit use. This is consistent with the fact that in dense areas, residents from different neighborhoods are less separated by physical space and may reflect the role of urban amenities, such as parks and public facilities, in facilitating diverse interactions (19). Experiences are also relatively less isolated in MSAs with higher income, more education, and lower unemployment. This could reflect a number of forces, including the role of social capital in reducing segregation (20). Experienced isolation is relatively lower where populations are younger, possibly reflecting the importance of schools and workplaces in reducing segregation. Finally, relative experienced isolation is negatively correlated with the Chetty et al. (21) measures of income mobility for both Black and White individuals, consistent with both diverse interactions increasing mobility and with areas that facilitate opportunity also promoting diverse interactions.

## Decomposing Experienced Isolation

### By Time

We first ask how experienced isolation varies over hours of the day. To do this, we restrict both exposures and the set of devices to all those that occur in a specific hour according to the MSA’s local time zone. Exposures are only estimated in geohash7s that are visited by devices that ping within that hour. For example, experienced isolation for 10 AM restricts our sample to pings that occur between 10 AM and 11 AM local time. After restricting the set of pings and devices, the estimation of experienced isolation is identical to our baseline measure.

**Fig. 4.**
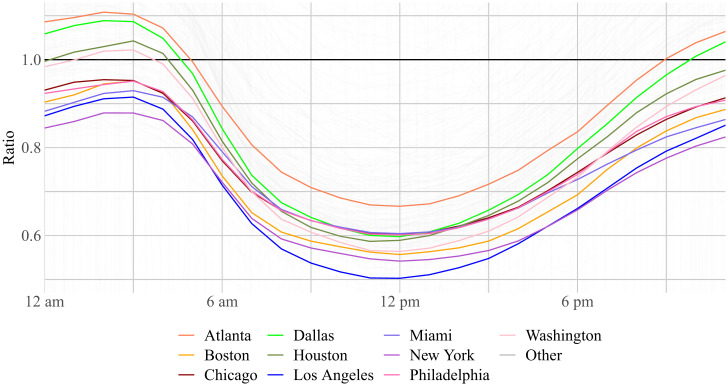
Experienced isolation relative to baseline by time of day. We plot the ratio of experienced to residential isolation in each hour of the day, highlighting the 10 most populous MSAs. Note that isolation can only be calculated for the devices active in a given hour, so the sample does change for each hour specification.

Fig. 4 plots experienced isolation over the course of the day, scaled relative to the level of residential isolation. The figure highlights the 10 most populous MSAs. The results are intuitive. Experienced isolation is lowest in the middle of the day as people move around and highest late at night as people withdraw into their homes. The ratio mostly differs in level between MSAs, and almost all MSAs share the same time profile.

### By Location

**Fig. 5.**
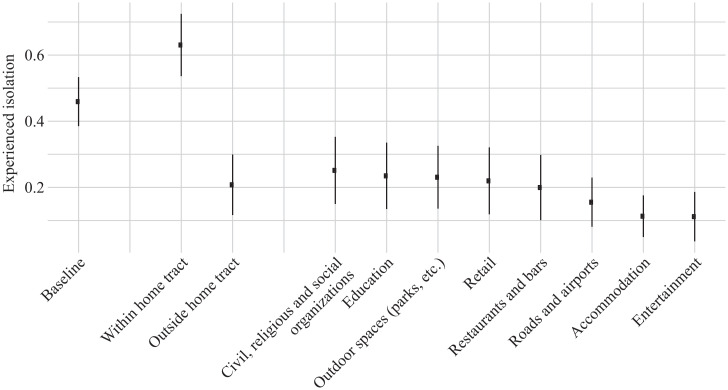
Experienced isolation relative to baseline by location. We plot the population-weighted mean experienced isolation in a particular feature and compare it with our baseline measure. Error bars show the population-weighted SD of experienced isolation across MSAs.

We next decompose experienced isolation by location. Much like restricting to pings within an hour, we restrict to pings that occur within a set of geohash7s of a particular type.^###^ These results are shown in Fig. 5. The leftmost point in the plot shows the average of our baseline measure of experienced isolation across MSAs, which includes all locations in our sample. The error bars in the plot indicate ±1 SD of the measure across MSAs.

The next two points in the figure show experienced isolation for locations within vs. outside of home census tracts. The results show that experienced isolation within home tracts (0.63 on average across MSAs) is higher than overall experienced isolation (0.46 on average) and actually higher than residential isolation (0.61 on average) (*SI Appendix*, Fig. S10 depicts experienced isolation within and outside home tracts). As discussed above, this result is not mechanical; experienced isolation within the home tract could differ from residential isolation in either direction, both because within-tract exposure is not uniform and because it includes visitors who live outside the home tract. In contrast, experienced isolation outside of home tracts is much lower, with an average of 0.21 across MSAs. Thus, time spent away from home is the key force reducing segregation relative to what the standard residential measure would suggest.

Fig. 5 summarizes the differences in experienced isolation for specific categories of features (*SI Appendix*, Fig. S13 depicts ping activity across features by WD/NWD designation). The baseline category contains all features, as well as time spent at home. Average experienced isolation in outdoor spaces, like parks, gardens, sports fields, and playgrounds, is only 50.3% of mean baseline isolation, and commercial establishments, like restaurants and bars, and retail stores have experienced isolation values that are only 43.5 and 47.8% of baseline isolation, respectively. Isolation is among its lowest in places of entertainment, like theaters (24.3% of baseline), and accommodations, like hotels (24.6% of baseline). *SI Appendix*, Table S9 shows summary statistics for experienced isolation across a wider set of feature types.

### By Race

Finally, we can decompose the differences in exposure that underlie the isolation index between WDs and NWDs. Experienced isolation is the difference between these groups in average exposure E[s(l,t)]. We ask how the experienced exposure relative to residential exposure differs by group. The results, which we present in *SI Appendix*, Figs. S11 and S12, show that the difference between experienced exposure and residential exposure is relatively small for WDs and much larger for NWDs. It also shows that NWDs’ experienced exposure varies much more across MSAs and across different feature types. This suggests that factors that reduce segregation away from home may have a particularly large impact on the experiences of NWDs.

## Robustness

*SI Appendix* reports a number of additional specifications probing the robustness of our main result. We provide detail on these specifications in *SI Appendix*, section S2 and show the results in *SI Appendix*, Table S10. They show that our main qualitative conclusions are robust to 1) excluding pings that are likely to occur while devices are commuting or traveling, 2) using alternative sources of demographic data, 3) excluding devices with home locations outside the MSA, 4) dropping the top 5% of devices in terms of the number of pings per day; 5) excluding pings occurring between midnight and 6 AM, and 6) using only the first ping emitted by a device in a given hour (so as to avoid overweighting hours with frequent pings). The final result in this table shows that we would overestimate experienced segregation if we used a naive estimator rather than the leave-out correction in Eq. **5**.

## Conclusion

The extent to which members of different groups are able to see, meet, and interact with one another can profoundly shape economic and social outcomes. Standard isolation indices capture such patterns under the assumption that people are uniformly exposed to others in their neighborhoods of residence. Our measure of experienced isolation builds on the activity space literature to relax this assumption and leverage location data to describe the exposures people actually experience as they move around over the course of their days.

We find that the exposure to diverse others that people actually experience is substantially greater than residential measures would suggest. People spend substantial time away from their home neighborhoods, and when they do, they are much more likely to encounter diverse others than they would at home. Commercial places, like restaurants and retail shops, are a particularly strong force pulling against segregation, while local amenities, such as churches and schools, tend to remain more segregated. One implication is that public goods that are tied to residential boundaries may deserve particular attention in efforts to combat segregation.

While experienced segregation and residential segregation are highly correlated across cities, the gap between them varies systematically, with relatively less experienced isolation in cities that are denser, wealthier, and more educated; that have greater use of public transport; and where income mobility is higher. These correlations do not allow us to draw any direct conclusions about either the causes or consequences of segregation, but they point toward factors that will be especially fruitful for subsequent research to investigate.

## Data Availability

Data cannot be shared. The data are based on device-level location data, which are sensitive. We have a data-sharing agreement that does not permit sharing.

## Footnotes

* Residential isolation based on our geographic definitions of WD and NWD is larger than the standard measure of residential isolation based on individual race. We discuss the reasons for this difference below.

^†^ Regarding policing and crime, Sampson and Levy (22) find that despite activity-based measures of segregation differing from residential measures, activity-based measures do not seem to mediate the influence of residential segregation on the degree of violence experienced by communities.

^‡^ Sunstein (24) writes that integrated physical spaces increase “the set of chance encounters with diverse others” and foster environments where “exposure is shared.” He argues that overhearing conversations while at a restaurant, a bus stop, or just walking down the street contributes to individuals’ understanding of diverse others and opens up opportunities for interaction.

^§^ Cagney et al. (11) in their review of activity space approaches identify explorations of such decompositions as an “enduring question” for the literature.

^¶^ Glaeser et al. (30) anticipate the value of such data. Blattman et al. (31) track police patrols in Bogotà, Colombia using GPS to estimate how increased state presence affects violent and property crime. Chen and Rohla (32) and Chen et al. (33) use GPS data to measure the effects of political polarization on the length of Thanksgiving dinners and to measure racial differences in waiting times at polling places, respectively. Davis et al. (7) use data from Yelp to measure the segregation of restaurants in New York City, finding that restaurants are less segregated than residential neighborhoods. Caetano and Maheshri (34) use data provided by the app Foursquare to quantify segregation by gender and by age in public places, and Phillips et al. (17) use geotagged tweets to build an index capturing the extent to which residents in each neighborhood of a city travel to all other neighborhoods in equal proportions.

^#^ Park and Kwan (36) define a notion of “multi-contextual segregation” that is closely related to our work in considering segregation over the varying geographic and temporal contexts of people’s daily lives.

^‖^ The geohash geocoding scheme divides the globe into grids of increasing fineness. Geohash1s divide the globe into 32 cells of equal size. Geohash2s divide each of these cells into 32 smaller cells and so on.

^**^ We use GPS as a shorthand for a variety of means used by smartphones to determine their physical location. These include cell phone towers and the identity of nearby Wi-Fi networks as well as the US GPS and the Russian Global Navigation Satellite System systems of satellites.

^††^ In our empirical analysis, we focus on the case where the groups *W* and *NW* partition the population, so that $1-e_{i}$ is individual *i*’s exposure to members of group *NW*. Our measure is also well defined in the case where some individuals in the population are neither in *W* nor *NW*. In this case, isolation where *e_i_* is the share exposed to *W* may be different from isolation had we defined *e_i_* as the share exposed to *NW*.

^‡‡^ This form of the isolation index is equivalent to Gentzkow and Shapiro (6). Much of the literature using the isolation index studies simply the exposures of a group, without taking their difference (9, 39, 40). Massey and Denton (8) provide a survey of other measures meant to encapsulate various qualitative aspects of segregation and motivate our decision to capture segregation by measuring exposure.

^##^ This theoretical measure of experienced isolation is related to the activity space approach of Wong and Shaw (12). Both consider the individual-level exposure of one group to another based on the share of individuals in locations of activity. Instead of theorizing in terms of discrete locations, we depart from their approach and consider the idealized continuous set of locations. With an abuse of notation, one can think of $\int_{t=0}^{1}l(i,t)$ as the activity space for individual *i*.

^¶¶^The random imputation strategy is equivalent to assuming that movement patterns are independent of individual race conditional on home geohash7. In simulations, we find that this tends to lead to a downward bias in estimates of experienced segregation.

^###^ If individuals never visit a geohash7 of the type in question, they are dropped from the sample.
